# Malaria in England

**Published:** 1919-09-20

**Authors:** 


### MALARIA IN ENGLAND.

Recently there was issued by the Local Government Board a series of reports and papers on malarial fever contracted in England in 1918, and it is noteworthy that the year showed a considerable decrease in the number of cases as compared with 1917. For some time past there have been repeated outcries that malaria threatened to become endemic in England as the result of returned cases of uncured infection from the military zones appearing in this country. The discussion has become at times involved with criticisms of the working of the Ministry of Pensions establishments, and although there is and has been undoubtedly some risk of a greater incidence in these islands, it appears likely that not a little misunderstanding of these matters exists in the public mind.

We read that, according to Dr. Newnham, of the London School of Tropical Medicine, there is little or no fear of a malarial epidemic occurring in England, and the fact remains that, although anopheline mosquitos are relatively common in the countryside, yet, as proved by the experiences of the last few years, they are by no means easily infected. Had this not been so, the presence of thousands of victims of the disease, returned from Mesopotamia, Salonika, and Africa, and in relatively close contact with the insects, would long ago have spread the infection broadcast.

During 1919, with the cessation of hostilities, the total number of malarial carriers transferred to England will doubtless be greater than in any year of the war. Frequently, and often unavoidably, many men are being demobilised who have recently had malarial parasites in their blood and who still suffer relapses at intervals. Instead of being still under military supervision, and in known areas, these cases are now scattered about the country, each in his own home; and if these homes are so situated and of such a type that they harbour anopheline mosquitos, one can be certain that to prevent the occurrence of new cases in them constant watchfulness and, when necessary, prompt action will be essential.

But this brings us to one of the most valuable contributions to the Report, namely, that by Colonel James, whose work in connection with malaria in this country certainly deserves to be more widely recognised than it has been. This observer has repeatedly noted that malarial outbreaks do not necessarily occur where either human "carriers" or the mosquitos are most concentrated, but rather in certain districts, and particularly certain types of dwelling. The local movements and habits of the English anophelines would seem to determine the spread in a high degree. Giving good evidence for his conclusions, Colonel James tells us that these mosquitos first seek a safe dark place to nest in and mature the eggs before depositing them, and during this stage bite vigorously. A bright, clean house without dark undisturbed corners is rarely visited, and therefore relatively, if not completely, safe. Thus the danger in this country seems localised to "malarial houses" rather than malarial districts, and if a "carrier" be present in one of these danger will threaten all the inmates. Yet in a clean house next door the people may be practically secure. If such be the true conditions, at once a fresh set of preventive but relatively simple measures present themselves, and are readily applicable in this country.

